# Indigenous Knowledge and Community‐Derived Counts Produce Robust Wildlife Population Estimates: Roosevelt Elk in Karuk Aboriginal Territory

**DOI:** 10.1002/ece3.72355

**Published:** 2025-10-19

**Authors:** Thomas Connor, Felipe Montealegre‐Mora, B. J. Saxon, Jessica Camarena, Daniel Sarna‐Wojcicki, Magali de Bruyn, Kendall L. Calhoun, Ciera C. Martinez, Emilio Tripp

**Affiliations:** ^1^ School of Natural Sciences Trinity College Dublin Dublin 2 Ireland; ^2^ Department of Environmental Science, Policy & Management University of California Berkeley Berkeley California USA; ^3^ Eric and Wendy Schmidt Center for Data Science and Environment University of California Berkeley Berkeley California USA; ^4^ Escuela de Física Universidad de Costa Rica San José Costa Rica; ^5^ Karuk Wildlife Team, Karuk Tribe Department of Natural Resources Orleans California USA; ^6^ Department of Ecology & Evolutionary Biology University of California, Los Angeles Los Angeles California USA; ^7^ Department of Wildlife, Fish & Conservation Biology University of California, Davis Davis California USA

**Keywords:** community based participatory research, conservation, elk, indigenous knowledge, traditional ecological knowledge, wildlife ecology

## Abstract

Community and citizen science‐based conservation projects have proliferated in recent years and hold promise for further incorporating Indigenous science and ways of knowing in conservation practice and decision‐making more broadly. Tribally led efforts that engage local community members in the data collection process can take advantage of generations of intimate place‐based knowledge for improved ecological monitoring, increased policy buy‐in, and ultimately more effective conservation practice in socio‐ecological systems. In this study, we detail community science research initiated and led by the Karuk Wildlife Team (KWT) to monitor Roosevelt elk ( *Cervus canadensis* , Karuk: *Ishyuux*) in their core winter range along the Klamath River in Karuk Ancestral Territory in northern California. Elk are a culturally and ecologically significant species that were extirpated from the area through overhunting after Euro‐American colonization in the mid‐19th century. They were reintroduced in the 1980s and are now annually harvested, necessitating effective monitoring. Encouraged by conversations with community members, the KWT began collecting detailed information on weekly elk sightings from community members along the Klamath River including date, time, number of elk seen, and approximate spatial coordinates (up to about ±200 m, accounting for the fact that elk groups could be observed from the highway on either side of the river). We fit an unmarked spatial capture‐recapture (uSCR) model in a Bayesian framework to community reports of elk from the winter of 2020–2021 to estimate their population density in our study area. We incorporated concurrent GPS monitoring data of three elk in this population to improve the spatial parameter, sigma, of our uSCR models. Our model estimated 0.56 elk/km^2^, a density that falls within expected ranges and mirrors the results of a camera trapping study we conducted in the same area. These results demonstrate the efficacy of a community‐driven monitoring protocol for harvested wildlife populations and the power of Tribally led science for effective conservation.

## Graphical abstract

We report on a tribally‐led study grounded on community observations to produce population density estimates for Roosevelt elk ( *Cervus canadensis* , Karuk: Ishyuux) in Karuk Aboriginal Territory, northern California. Elk are a culturally and ecologically significant species that were extirpated through overhunting after the Euro‐American colonization in the mid‐19th century, and since the 1980s they have been reintroduced and annually harvested, necessitating effective monitoring. We use unmarked spatial capture‐recapture models and Bayesian inference based on Markov chain Monte Carlo in order to infer population density from community reports and telemetry data.

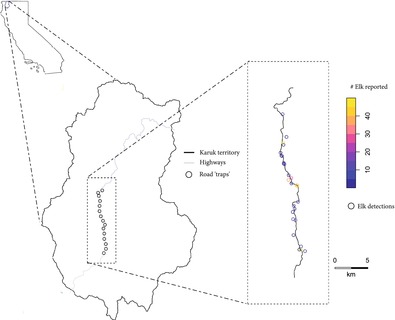

## 1 Introduction

Wildlife and environmental management and research should actively include Local Ecological Knowledge (LEK)—the knowledge of those who live and co‐exist within the local landscape. LEK knowledge can be incorporated at different points in the research cycle; from community‐collected approaches, limited to data collection, to community‐driven approaches, where the community sets the research agenda, to ideally participatory science practices, in which the community is engaged at every stage. When the LEK includes Indigenous communities, the nature of involvement must be further defined to recognize and respect Indigenous knowledge systems including Traditional Ecological Knowledge (TEK), which refers to environmental understanding developed through long‐term interaction with and use of local resources. Community‐collected, community‐driven, and participatory science can align closely with the principles of TEK, Indigenous science and Tribal efforts in monitoring and interaction with wildlife (Ramos 2022). All these types of approaches emphasize a place‐based perspective that builds knowledge from a local, bottom‐up framework (Haywood et al. 2016; Artelle et al. 2021). However, TEK is seldom fully woven into scientific conclusions in a manner that authentically values and blends both knowledge systems (Berkes 2017). In some instances, the goals of community‐engaged and scientific approaches can come into conflict with Indigenous methodologies (Benyei et al. 2021, 2023). For example, the push towards open science through large, publicly available databases undermines Indigenous data sovereignty. Community‐based participatory research and Indigenous methodologies originate from distinct epistemic lineages and histories of practice. Each approach defines what constitutes a community differently and involves unique regimes of participation and interaction between professional researchers and community members. Combining these differences shapes the power dynamics in research, influencing the level of community control, authority, and oversight in the process (Nadasdy 1999, 2007; Pocock et al. 2018; Benyei et al. 2021).

Elk ( *Cervus canadensis* , Karuk name: *Ishyuux*) are both a keystone species and a culturally significant one to the Karuk Tribe in Northern CA, playing an essential role in the local socio‐ecological systems. Elk are a focal species for Tribal‐led fire and fuels management and forest ecosystem restoration activities in Karuk Aboriginal Territory (KAT) in the middle Klamath Basin (The Karuk Tribe 2018, 2019; Connor, Wildlife Division, et al. 2022). Karuk elk habitat and herd management is a priority for the Karuk community, as it furthers efforts to restore local ecosystems and watersheds, expand access to cultural foods and fibers, support community health and food sovereignty, revitalize cultural practices, and enhance tribal sovereignty. Since time immemorial, the Karuk People have actively stewarded elk populations, continuously monitoring their numbers across both short‐term and generational timescales. Traditionally, these TEK practices involved direct interaction with elk herds, where assessing population size was a collective effort based on observation, and knowledge was passed down through socially guided practices. These practices have evolved over time, making Karuk TEK inseparable from the place‐based practices in which it is embedded, as well as from the broader bodies of knowledge our community partners hold about wildlife and their relationships with the ecosystems they inhabit. The role of TEK in Karuk‐led elk population management now blends traditional knowledge passed down through generations and modern monitoring and statistical modeling methods originating from western scientific research (Arnold et al. 2018).

Given the complexity of managing wildlife populations, combining TEK with modern monitoring and modeling methods provides a more comprehensive foundation for conservation practice. Modern research approaches include the detection of animal presence or absence from signs of their activity (e.g., tracks, scat), counts from ground and air surveys, camera trapping, and the capture and recapture of individuals through visual or genetic marking (Waits and Paetkau 2010; Gilbert et al. 2021). To glean the most inference from these data, a variety of statistical methodologies have been developed to estimate population sizes, ranging from binomial models of only species detection–non detection information to capture–recapture models of identified individuals (Royle and Nichols 2003; Royle et al. 2009; Sutherland et al. 2019). Most of these methodologies require substantial field effort to collect data, and some require extensive laboratory‐based work and associated costs to process data, generally limiting the potential sampling area, effort, and duration of surveys (Chandler and Clark 2014; Furnas et al. 2018; Bach et al. 2022). In this paper, we present a study that offers a strategy to incorporate TEK informed and Indigenous led observational data collection through the entire data life cycle to estimate Elk population size.

If grounded in Indigenous‐led protocols, collecting data from the community draws on TEK expertise while building trust between Tribal staff and the broader tribal community. Tribal protocols vary by community but are important for holding researchers accountable to Tribally defined standards for ethical and equitable practice, including Tribal review of research processes and outputs, sharing in benefits such as grant funding or authorship on publications, addressing past and ongoing ecological and social harms, giving back to the community and landscape, educating and empowering youth, and ensuring Tribal data sovereignty (Karuk Tribe Department of Natural Resources, n.d.; Sarna‐Wojcicki 2014; Diver and Higgins 2014; Sowerwine et al. 2019; Oberholzer Dent et al. 2023; Smith, Diver, and Reed 2023; Hudson et al. 2023). If combined appropriately through Tribal‐led processes, community science and community‐engaged research can be braided with emerging methods in wildlife research, allowing for broader applications, such as across an entire region or territory. Furthermore, it can help identify locally validated practices for management and restoration, contributing to the resolution of conservation conflicts and the development of culturally appropriate land‐use regulations and stewardship practices (Fernandez‐Gimenez 2000; Babai et al. 2015).

To explore the potential for community‐collected data to contribute to wildlife conservation and management while inviting contributions of TEK, we engaged with and designed the Community Reports Program that collected community reports of elk sightings in a portion of KAT in northern California as part of a wider study of elk ecology in the region. The Community Reports Program was led and designed by the Karuk Wildlife Team (KWT), an indigenous‐led group committed to the revitalization of wildlife populations and habitats in Karuk lands. An important motivation for the wildlife program in conducting this outreach was to build trust with the Karuk local community and enroll them in observing and caring for wildlife in Karuk lands. Encouraging community members to share their TEK requires a solid foundation of trust and deep relationships between the broader Tribal community and the Indigenous‐led wildlife program. To gain greater inference, we explored the potential to fit spatial count models to these data to estimate elk population density throughout the area.

## 2 Methods

### 2.1 Study System

**FIGURE 1:**
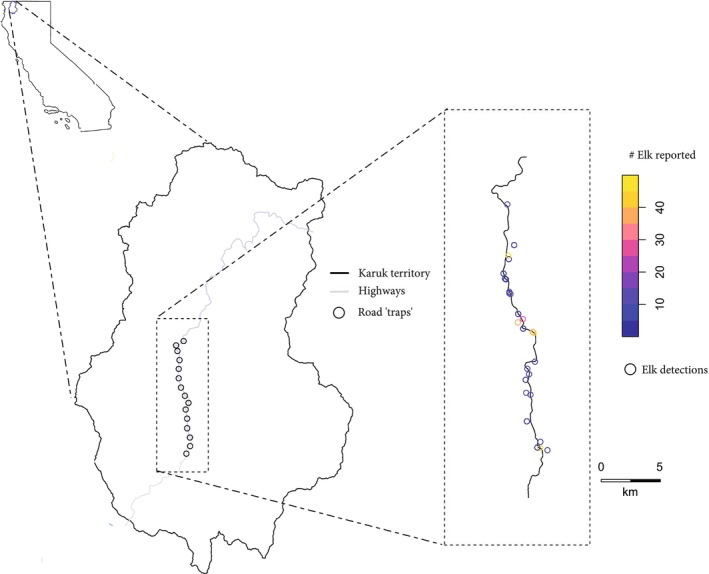
Map of study system, with our road “trap” locations within KAT inset on a map of California. The final panel depicts the elk sighting report locations, with the increasing numbers of elk reported in the sighting represented by warmer colors. Highway 96, which served as our sampling corridor, spatially follows the Klamath River.

We conducted our research in the core of KAT in far northern California, USA (Figure 1). The Karuk Tribe and their lands represent a continuously settled culture from time immemorial, and despite extensive persecution after Euro‐American colonization, they were not forcibly displaced from their ancestral territory (Salter 2003). This territory is part of the floristically diverse Klamath ecoregion that features a Mediterranean climate with warm, dry summers and cool, wet winters (Sawyer 2006). The rugged mountains and river valleys range from about 123 m to over 2500 m in elevation. The core of KAT straddles the Klamath River, which encompasses much of the area's elk winter habitat falling below 780 m in elevation (Allison et al. 2007). The forests throughout the area feature a dominant coniferous overstory consisting of Douglas‐fir ( *Pseudotsuga menziesii var. menziesii* ), incense cedar ( *Calocedrus decurrens* ), sugar pine ( *Pinus lambertiana* ), Jeffrey pine ( *Pinus jeffreyi* ), and white fir ( *Abies concolor* ) (Taylor and Skinner 2003). Temperatures range from lows of around 0°C in December/January to highs of over 30°C in August.

Roosevelt elk is a culturally important species that traditionally made up an important source of food, clothing (hides), regalia, and implements for the Karuk, and were valued for their role in shaping ecosystems (Karuk Department of Natural Resources 2011; Norgaard 2014c). Due to fire suppression, habitat loss, and hunting for meat and hides by Euro‐American colonists, nearly all elk were extirpated from the Karuk Tribe's ancestral territory as early as the 1870s (Harper et al. 1967). Beginning in 1985, six Roosevelt Elk from Redwood National Park were reintroduced into Elk Creek in Karuk Aboriginal Territory by California Department of Fish and Wildlife (CDFW) and US Forest Service/Klamath National Forest with the support of Karuk tribal community members. Several Roosevelt Elk were also reintroduced a few miles north of Somes Bar, California in Karuk Territory in 1985 through a CDFW–USFS collaboration (Personal Communication, B.J. Saxon, USFS staff 2018). By 1996, 232 Roosevelt elk had been reintroduced into Klamath National Forest and the Marble Mountain wilderness by the US Forest Service and California Department of Fish and Wildlife (CDFW). There now exist at least four elk herds in the Marble Mountains, with a total population size that has been estimated at around 3000, and there is a need for improved estimates and monitoring over time (*Elk Conservation and Management Plan, California Department of Fisheries and Wildlife* 2018; Tripp et al. 2022). Building on decades of work restoring elk populations, beginning in 2018, the KWT developed methods for studying local elk populations in collaboration with the Karuk Department of Natural Resources and academic partners, following Tribal research protocols. To complement camera arrays, fecal DNA sampling, and collaring efforts focused on elk winter range in KAT, the KWT developed methods for collecting community reports from the broader Karuk community, who are both passionate and knowledgeable about local wildlife populations across KAT (Tripp et al. 2022; Connor, Tripp, et al. 2022).

### 2.2 Data Collection and Dissemination

Based on the LEK of the KAT, the Karuk Wildlife Supervisor proposed to the newly established KWT that conducting early morning elk counts along the Klamath River Corridor, the wintering habitat of the Karuk Roosevelt Elk, would yield the most accurate population estimates. This suggestion led to the implementation of Drive Surveys, conducted on the mornings following new and full moons. This activity drew on TEK from Native hunters grounded in local empirical observation and experience related to elk habitat use and movement across the landscape throughout the seasons. The success of these surveys inspired the design of the Community Reports Program, a broader initiative to gather wildlife observational data, including elk data from Karuk community members, who regularly commute through the winter range along Highway 96, extending from Orleans to Happy Camp and even up to the I‐5 corridor. This program aimed to develop a more robust dataset on the local Karuk wildlife population and drew from the community's local and place‐specific knowledge and inherited values of sustainable stewardship of resources throughout the entire data life cycle. Throughout, we focused on ensuring that our work aligned with the Karuk community's goals and values; this was facilitated by using the *Practicing Pikyav* Tribal oversight protocol from the onset of the project.

Beginning in December of 2018, the Community Reports Program allowed a more systematic way to gain associated wildlife counts from community members. The reports were solicited from Karuk Department of Natural Resources co‐workers who traveled the State Highway 96 frequently, which parallels the Klamath River and thus the core of the winter range of elk in the area. The Community Reports Program was also advertised in the Karuk Tribe quarterly newsletter to expand the potential contributor set beyond those working at the Karuk Department of Natural Resources (KDNR). KWT also set up a booth at the Tribal reunion and printed postcards with contact information asking for wildlife observations. Reports were taken both orally and through written responses with an explicit ask for consent to use the data for research purposes. For each report, the date, method of collection (phone, in person, social media, etc.), community member, report collector, number of wildlife observed and their age class/sex (if observed), and location were collected. We used the report cases in which elk sightings were observed for this study. While not explicitly collected as information in the reports, the reports also functioned to give opportunities for the community to share knowledge not easily quantified, including LEK and TEK knowledge, and stories of wildlife in the regions. This process built trust and encouraged continued engagement. The data used in this study were location and sightings of elk in the region; if longitude and latitude coordinates were not explicitly reported, these coordinates were estimated using the location descriptions given by the community member and the Avenza mapping app (Avenza Systems Inc. n.d.). The Community Reports Program has been active for 7 years at the time of writing of this paper, with a total of 226 total elk sightings coming from 46 unique reporters. In this study, we subsetted the elk sightings to a single geographic region with camera traps, with the aim of allowing future comparative studies between community report counts and camera trap counts. We focused on 3 months of the 2020/2021 winter season (December–February) in which the elk range intersects the lower altitude regions near Highway 96. In this geographic and seasonal subset of the data, a total of five individuals submitted 28 community reports on elk population counts, with one individual providing substantial elk sighting observations. The KWT disseminates details and results of research and the Community Reports Program in community meetings with dinner. In addition, the KWT brings this work into the K‐12 local education programs with visits to classrooms, curriculum development, and a summer internship program.

In addition to data collected from the Community Reports Program, we collected complementary data during this time period. Among these were GPS telemetry data from three collared elk individuals during the study period. Elk were captured using eight clover traps placed in meadows adjacent to the Klamath River and baited with alfalfa in the fall/winter of 2020. One young male was sedated using a (CDFW‐administered) tranquilizer drug consisting of Butorphanol tartrate, Azaperone tartrate, and Medetomidine hydrochloride (BAM) attached to the end of a “poke pole,” while the three females were handled without drugs. Each elk was fitted with Vectronic Vertex Lite 2D GPS collars set to transmit location fixes every 2 h (Vectronic Aerospace GmbH, Berlin, Germany). All elk captures and handling were conducted under capture plans approved by the Karuk Tribe in coordination with the California Department of Fish and Wildlife.

### 2.3 Statistical Modeling

To convert the community report data into a format conducive to modeling, we created a series of 15 artificial “traps” (i.e., sampling locations) spaced 1.95 km apart along Highway 96 (Chandler and Royle 2013). To estimate population size for a single winter season, we selected community reports in which elk were sighted in the 15 weeks between December 22, 2020, and April 1, 2021. We chose this winter because community report collection had been occurring for almost 2 years and was thus well established, and we had three active GPS collars deployed and collecting data on elk movements in that time period. We also limited the study period to these 15 weeks to reduce violations of the model assumption of population closure (limited new individuals and/or mortality of individuals over the period). Elk counts were binned to the nearest trap location along Highway 96. While there was likely variation in sampling effort between different sections of the road, we could not reliably measure these variations and thus we assumed equal effort across the length of the road.

To estimate population size, we used an unmarked spatial capture‐recapture (uSCR) model fit to the community report‐derived count data (Chandler and Royle 2013). This class of model uses the spatial autocorrelation of counts to predict the density of animals per square kilometer in the study area. For this, a standard recommendation is for camera traps in uSCR studies to be spaced 1–3 times the typical radius of an individual's home range. We informed the spacing between artificial traps in our study on elk movement capacities, with traps being approximately 1.95 km apart (Ramsey et al. 2015). We also used elk movement capacity to inform a road buffer distance of 4 km to capture potential elk activity centers for individuals observable from the road (Connor, Wildlife Division, et al. 2022), creating an effective study area of 336.26 km^2^. (Chandler and Royle 2013). Model assumptions include independent animal activity centers and uncorrelated detections, both of which were likely violated to some degree given elk herding behaviors.

We used an uninformative prior on all parameters except for the space‐use parameter, whose prior was informed by the movement data gathered from three elk with GPS collars to be

(1) $$\sigma \sim \text{Normal}\left(\text{mean}=1600\;m,\mathrm{st}.\;\text{deviation}=200\;m\right)$$

Posterior distributions on model parameters were obtained using two Markov Chain Monte‐Carlo (MCMC) chains with the R package R‐Nimble (de Valpine et al. 2017) with 25,000 burn‐in steps and 100,000 steps in total. We used the Gelman‐Rubin R‐hat statistic (< 1.1) and a visual inspection of trace plots to assess adequate mixing of the chains and model convergence (Gelman and Rubin 1992).

## 3 Results

A total of 28 community reports were collected during the winter 2020/2021 study period. The number of reported elk varied between 1 and 50, with a mean of 15.18 and a median of 9.50. Reports were temporally spread across the entire study period but with a peak in late January/early February and more reports in the late winter compared to December/early January. These 28 reports came from five different community members, with all except one submitting more than one report. A single community member was especially prolific, submitting 19 reported elk sightings across the study period.

Three elk (one male and two females) were captured and fitted with GPS collars prior to our study period. All elk had consistent GPS collar location fixes throughout the study, and we subsampled 1000 GPS locations per individual to inform the sigma parameter. Fitting model (2) to the GPS data, we obtained an estimate $\sigma_{\mathrm{GPS}}\approx 1600\;m$ which we used to inform our prior on the space use parameter σ in model (1), see Equation (1).

**FIGURE 2:**
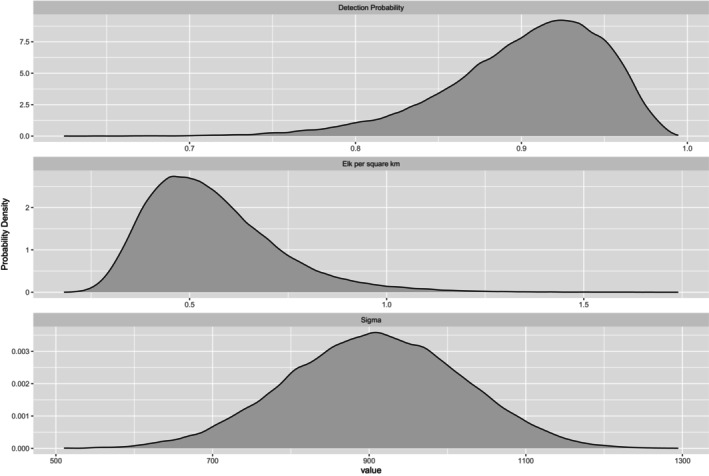
Posterior distributions of the elk population density, sigma (space use), and probability of detection parameters from our Bayesian unmarked spatial capture‐recapture model of community reports of elk in Karuk Aboriginal Territory. In this model, GPS collar telemetry data were used to define a prior distribution informing the sigma (space use) parameter.

A visual inspection of the MCMC trace plots showed good mixing, and R‐hat values for all parameters were < 1.1 indicating convergence. The posterior distributions for parameters obtained from our uSCR model fit to community report data are visualized in Figure 2. Associated summary statistics are collected in Table 1. The elk population density was estimated at 0.56 elk/km^2^ (95% CI: 0.32–0.96 elk/km^2^), while the detection rate was estimated to be 0.90 (95% CI: 0.79–0.97). Space use was estimated to be 906 m (95% CI: 689–1119 m).

**TABLE 1:** Posterior results of our Bayesian unmarked spatial capture–recapture model of community reports of elk in Karuk Aboriginal Territory.

Parameter	Mean	SD	2.5% CI	97.5% CI
Density (elk/km^2^)	0.56	0.17	0.32	0.96
$p_{0}$ (detection rate)	0.90	0.05	0.79	0.97
σ (space use, meters)	906.75	110.66	688.92	1118.79

## 4 Discussion

Our results showcase the potential for harnessing community‐derived wildlife data in Tribally led wildlife research and management initiatives. Engaging the community in wildlife research not only helped the KWT nurture relations with the broader Tribal community, it complemented other research methods and helped build more robust data sets about local wildlife populations. The KWT provided leadership from within the community and acted as a liaison between academic researchers and the broader Tribal community. The overall research project was grounded in Tribal research protocols and oversight processes, and the methods were designed in full collaboration with the Karuk community and its representatives at KWT and KDNR. The community was therefore able to trust that the research was being conducted in the best interest of the broader community and that the data was being collected, stored, analyzed, and stewarded safely by the KWT.

We were able to fit uSCR models (not presented here) to only the community report‐derived elk counts without supplementary telemetry data. However, these models featured slight bimodality in which uncertainty in the space use parameter drove different possible values of population density. The use of concurrently collected GPS collar telemetry data allowed us to define an appropriate prior distribution for a better estimate of the sigma (space use) parameter, helping us arrive at more robust estimates of population density. The integration of detailed animal space use information into spatially explicit population models, either by informing prior distributions (Ramsey et al. 2015; Furnas et al. 2018) or by helping to directly estimate space use within the model (Ruprecht et al. 2021), has been shown to increase the precision of parameter estimates. We also explored fully integrating the telemetry data into the model, that is, using $\sigma_{\mathrm{GPS}}=\sigma$ in Equation (1) and estimating the joint likelihood using the community report data and telemetry data simultaneously. However, this seemed to hamper the model's convergence, ultimately leading to unrealistically low density estimates. We suggest further research is needed to explore the direct integration of these data types within a model.

The posterior estimate obtained for σ was lower than the value suggested by the GPS data (σ=907m compared to 1600 m). This downward trend in sigma estimates has been previously observed when fitting uSCR models in Connor, Wildlife Division, et al. (2022) and Ramsey et al. (2015). Generally, there is very little space use information in unmarked counts of animals, and we suspect the large aggregations of elk likely increase the estimated spatial autocorrelation in detections. Group dynamics could account for some part of this discrepancy (Emmet et al. 2022; Hostetter et al. 2022; Sun and Cole Burton 2024)—the base uSCR model ignores group dynamics, and detection rates are treated as independent for each elk in the simulated population. Violation of this assumption may have resulted in biased low density estimates, in particular (Sun and Cole Burton 2024). The incorporation of group and animal movement dynamics within uSCR models (McClintock et al. 2012, 2022) could allow for a more robust integration of community reports data with telemetry data. Moreover, these extensions of uSCR models could enable more connections between Karuk TEK of elk dynamics and the inference models used. This significant methodological contribution was outside of this paper's scope, however, and is left for future work.

While we cannot know the true population density of elk within our study area, our model results fall within the range of typical density estimates of wild elk in Western North America, though some western populations exist at much higher densities (Hebblewhite et al. 2002; Smith, MacNulty, et al. 2023). Additionally, we have made previous estimates of elk population density in our study area based on spatial count data derived from camera trapping and spatial capture–recapture (SCR) data derived from elk scat sampling, and our estimate falls within those ranges. Specifically, the density of 0.56 elk/km^2^ we estimated with our uSCR model of community report‐derived elk data in this study corresponds very closely to our previously published estimate of 0.57 elk/km^2^ from a uSCR model of camera trapping‐based elk counts (Connor, Wildlife Division, et al. 2022). Given the concordance of these results, we find it likely that the data collection and modeling methods described in this study resulted in fairly accurate estimates of elk population density. That said, our SCR model using elk scat sampling data resulted in a higher estimate of nearly 1 elk/km^2^, suggesting that both the camera trapping and community report‐based uSCR models may have density estimates that are biased low. A camera trapping study using different methods conducted across elk summer range in a much broader encompassing area estimated much lower densities (0.07 elk/km^2^, Moriarty‐Graves et al. 2023). While it is difficult to directly compare these estimates as the study areas varied substantially, we are confident that our results better represent the densities of elk populations wintering around the Klamath River in KAT, given our large local sample size of counts and scat‐based spatial capture–recaptures (Connor, Wildlife Division, et al. 2022; Connor et al. 2023). From a conservation and management perspective, estimates biased low are typically less concerning than estimates that are biased high due to the risk of overharvest. Still, our results and other literature suggest that at least the partial marking or recognition of individuals and integration of those data into population models would help improve estimates and, in this case, reduce a likely negative bias (Chandler and Royle 2013; Connor et al. 2023).

Success in simultaneously developing a culturally meaningful framework for elk population estimation and expanding trust among Tribal community members serves to reinforce the core values of the Tribal community and pull more engagement for future, longer‐term work. There are further potential community data that could be collected to improve our model by better informing the detection process. Specifically, documenting how many community members were actively looking for elk along which sections of road at what times would allow us to account for uneven effort and detection probabilities at each road “trap” over the course of the survey. Collecting information about the approximate distances to each elk group that community observers were at each sighting would allow the implementation of alternate modeling frameworks such as distance sampling, which may suit these group‐based data well (Schmidt et al. 2012). This level of detail would require further buy‐in from community members and increased logistical burden on Karuk wildlife practitioners, but may improve model accuracy and precision. The KWT is actively working on these advancements by sharing results with and seeking feedback from community members through Tribal newsletters and in‐person interactions. With sufficient funding resources, future work could include developing an application and/or website dedicated to collecting community wildlife observations for a broader range of species and interactively sharing analytical results. These developments would also likely aid in expanding the sample of community members participating in wildlife science and decrease the variance in the number of reports across community members by reducing the difficulty in participating. Finally, expanding the sample size of GPS collars on elk in the region would allow better estimation of movement behaviors and allow for potentially including estimates of sightability (i.e., the rate at which observers detect animals we know were in an area) to further increase the robustness of the model.

## 5 Conclusion

In addition to deeply engaging local stakeholders and community members, the collection and use of data from The Community Reports Program, when combined with modern detection and survey methods, has the potential to generate robust data and models for Indigenous‐led wildlife management practices. This supports more sustainable management of their resources (Berkes 2007). While state‐level management of wildlife in the United States often harnesses data derived from hunter success rates and harvested animals, the broad statewide scale of these methods typically means that implicated individuals are not actively engaged beyond mandatory reporting policies in the collection and use of those data (Van Buskirk et al. 2025). Conversely, our study was initiated by enthusiastic community members discussing elk sightings with the KWT, combined with the vision and effort by KWT members to further encourage, collect, and collate those community reports into a comprehensive dataset. This paper demonstrates the power and potential of combining different ways of engaging an Indigenous community and their knowledge in wildlife research. Through the Practicing Pikyav Tribal oversight protocol, the Tribe was able to ensure from the very beginning that the research methods were aligned with community values and priorities, that sensitive cultural information was being used and shared appropriately, and that ultimately the research was serving the broader community's needs. With this protocol in place, a foundation of trust was built between the research team and the community such that the community was able to share knowledge with the research team and ultimately a global audience through publication. In addition to building trust with the local community and a forum for sharing wildlife knowledge with the KWT in a culturally appropriate way, this research helps build towards a method for understanding elk population dynamics across time and space to inform landscape stewardship for elk going forward. With the KWT leading the research and by following Indigenous research protocols, community sourced data was used to fit a statistical model and make it both actionable and valuable to (Indigenous) wildlife practitioners and the broader Indigenous community.

Understanding elk population dynamics is central to better managing the landscape to enhance their habitat, particularly in their winter range habitats, which are the most limited and degraded seasonal habitats in Karuk territory. Enhancing the suitability of habitats and carrying capacity of the landscape to support elk populations will in turn promote healthy elk herds in Karuk lands in perpetuity. Better stewardship of Karuk lands for elk herds helps the Karuk wildlife team fulfill their mission to “enhance, protect, conserve, and restore the ecological processes that the wildlife depend on in Karuk Aboriginal Lands, to support the physical, cultural and spiritual well‐being of the Karuk community and to continue this ancestral legacy of nurturing and protecting the traditional relationships of respect and reciprocity with our wildlife family for the future generations to come.”

In Indigenous‐led approaches, community members are not just contributors of scientific data; they are fellow stewards of the landscape and component habitats, and invaluable sources of TEK and management practices (Norgaard 2014a; McAllister et al. 2023). TEK, though deeply rooted in history, is dynamic, evolving as each generation adapts it to contemporary contexts (Polfus et al. 2014; Berkes 2017; Peoples Programme 2020), including the incorporation of modern scientific methodologies and technologies. A central goal of Indigenous‐led and TEK research is to strengthen traditional communities, their knowledge systems and their place‐based relationships by promoting local activities and spaces where knowledge is produced, exchanged, governed, and validated. These goals ensure that TEK remains adaptive to change and responsive to place‐based priorities for ecocultural revitalization (Norgaard 2014a, 2014b; Reyes‐García et al. 2019; Biró et al. 2020). As Reid et al. (2024) notes, Western research approaches can be problematic for Indigenous communities due to the past and present harms caused by colonial science and land management practices to Indigenous lands and people (Sowerwine et al. 2019; Eichler and Baumeister 2021; Klein et al. 2022; Reid et al. 2024). The authors call for ethical and equitable approaches to conducting ecological research “in a good way” with the Indigenous communities who have lived on and cared for their lands since time immemorial (Reid et al. 2024; Ogar et al. 2020; Fisk et al. 2024).

The co‐creation of the Community Reports Program by community members and Karuk resource stewards highlights the powerful potential for more effective management at the intersection of community science and Indigenous TEK. By actively utilizing community‐derived data collected through active engagement for wildlife monitoring, the KWT is explicitly considering community members as valuable sources of knowledge and integral to the socio‐ecological system. Community members, in turn, are empowered as resource stewards themselves, and by taking an active role in wildlife monitoring, are likely to better understand and support any management or conservation action taken as a result. Indigenous communities retain place‐based knowledge, cultural practices, and belief systems that are based on thousands of years of caring for their lands and are therefore best positioned as stewards of wildlife in Indigenous territories. More funding, resources, policy, and legal support are needed to enable Indigenous communities to play leadership roles in wildlife research and management. Academic researchers and institutions can play an important role in supporting Indigenous‐led and community‐engaged wildlife research, provided protocols are in place to ensure equitable research partnerships and that the research is ultimately being conducted by and for Indigenous communities to benefit the wildlife relations of Indigenous lands.

## Author Contributions

**Thomas Connor:** formal analysis (lead), investigation (equal), methodology (equal), software (supporting), writing – original draft (equal), writing – review and editing (equal). **Felipe Montealegre‐Mora:** formal analysis (lead), investigation (equal), methodology (lead), software (lead), writing – original draft (supporting), writing – review and editing (supporting). **B. J. Saxon:** conceptualization (lead), data curation (lead), investigation (equal), methodology (lead), supervision (lead). **Jessica Camarena:** conceptualization (lead), data curation (lead), investigation (equal), methodology (lead). **Daniel Sarna‐Wojcicki:** investigation (equal), supervision (lead), writing – original draft (equal), writing – review and editing (equal). **Magali de Bruyn:** conceptualization (equal), software (supporting), writing – original draft (lead), writing – review and editing (lead). **Kendall L. Calhoun:** formal analysis (supporting), writing – original draft (supporting), writing – review and editing (supporting). **Ciera C. Martinez:** conceptualization (equal), writing – original draft (lead), writing – review and editing (lead). **Emilio Tripp:** conceptualization (lead), data curation (lead), formal analysis (supporting), funding acquisition (lead), investigation (equal), resources (lead), supervision (lead).

## Conflicts of Interest

The authors declare no conflicts of interest.

## Supporting information


**Data S1:** ece372355‐sup‐0001‐Supinfo01.zip.

## Data Availability

To honor the trust that has been afforded to this team by the members of the Karuk community, and following the CARE principles for indigenous data sovereignty, the data and code used to produce the results in this paper are not publicly shared. All the necessary code and data to reproduce this paper are provided as Supporting Information for the review process.
